# Design and Realization of an Efficient Large-Area Event-Driven E-Skin

**DOI:** 10.3390/s20071965

**Published:** 2020-03-31

**Authors:** Florian Bergner, Emmanuel Dean-Leon, Gordon Cheng

**Affiliations:** Institute for Cognitive Systems (ICS), Technische Universität München, Arcisstraße 21, 80333 München, Germany; dean@tum.de (E.D.-L.); gordon@tum.de (G.C.)

**Keywords:** tactile sensing, e-skin, large-area skin systems, event-driven systems, send-on-delta principle

## Abstract

The sense of touch enables us to safely interact and control our contacts with our surroundings. Many technical systems and applications could profit from a similar type of sense. Yet, despite the emergence of e-skin systems covering more extensive areas, large-area realizations of e-skin effectively boosting applications are still rare. Recent advancements have improved the deployability and robustness of e-skin systems laying the basis for their scalability. However, the upscaling of e-skin systems introduces yet another challenge—the challenge of handling a large amount of heterogeneous tactile information with complex spatial relations between sensing points. We targeted this challenge and proposed an event-driven approach for large-area skin systems. While our previous works focused on the implementation and the experimental validation of the approach, this work now provides the consolidated foundations for realizing, designing, and understanding large-area event-driven e-skin systems for effective applications. This work homogenizes the different perspectives on event-driven systems and assesses the applicability of existing event-driven implementations in large-area skin systems. Additionally, we provide novel guidelines for tuning the novelty-threshold of event generators. Overall, this work develops a systematic approach towards realizing a flexible event-driven information handling system on standard computer systems for large-scale e-skin with detailed descriptions on the effective design of event generators and decoders. All designs and guidelines are validated by outlining their impacts on our implementations, and by consolidating various experimental results. The resulting system design for e-skin systems is scalable, efficient, flexible, and capable of handling large amounts of information without customized hardware. The system provides the feasibility of complex large-area tactile applications, for instance in robotics.

## 1. Introduction

The sense of touch plays an essential role in our lives and allows us to safely interact and control our contacts with our surroundings. The sense of touch not only allows us to characterize and evaluate contacts, but it also allows us to locate contacts. It provides us with a tactile image of our interactions with the world. The human brain represents this tactile image in the somatosensory cortex combining proprioceptive information with cutaneous information and thus assembling an internal model, that is, the homunculus that associates the postural information of the body with the spatial location and tactile information of the skin receptors [1,2].

A deeper study of the sense of touch reveals its fundamental differences in comparison to our other senses. These differences eventually break down to two facts. The sense of touch is a highly *distributed* sense. The sense of touch spreads out its 5 million cutaneous receptors [2] (mechanoreceptors, thermoreceptors, nociceptors) through the whole body in large areas up to around 2 m${}^{2}$ [3] and conveys tactile information through around 1.1 million ascending nerve fibers [4] to the somatosensory cortex. In contrast to the sense of touch, vision is a very concentrated sense. The human eye accommodates approximately 137 million receptors (130 million rods and 6.5 million cones per retina) [5] and approximately 1 million nerve fibers [6] in an area of around 1100 mm${}^{2}$. In comparison, the sensing area of the human skin is around 1000 times larger than the sensing area of both eyes. Consequently, the term *large-scale* can be attributed to large resolutions in vision and to *large-areas* in tactile sensing. Secondly, the sense of touch acquires its information through *physical contacts*. In contrast to vision or audition which protect their receptors against physical contacts, the sense of touch depends on these contacts with the environment to acquire information. Tactile receptors cannot acquire tactile information through distant observations, they need to access the information in the area of the contact. These two facts profoundly impact how the sense of touch organizes sensing in nature [7] and thus provide the guidelines to effective electronic skin (e-skin) designs [8,9].

The developments of e-skin currently focus on two different kinds of skins similar to the two found in humans [10,11]. One skin is mainly located in the inner sides of the hands and the foot-soles while the other skin covers the remaining parts of the body. The skin of our hands and foot-soles covers rather small regions and targets very high spatial resolution, supersensitive sensing, shear-force, and vibration sensing, and slip detection [10]. Research towards realizing this kind of skin in technical systems deemphasizes the challenges of distributed sensing systems and focuses on high sensing density and the challenges connected with supporting physical contacts. Such as the development of fingertip e-skins that have been investigated in the works in References [12,13,14]. Developing e-skin to cover large areas emphasizes the distributed nature of the sense of touch. While large area skin may slightly deemphasize high spatial resolution, it has to specifically focus on efficient and feasible methods to deploy, connect, and determine the poses (location and orientation in 3D space) of a *large number* of spatially distributed *tactile* sensors over *large areas*. We define these e-skin system as large-area skin systems (LASSs).

The sense of touch employs specialized receptors for sensing mechanical, thermal and noxious (potentially dangerous/destructive) stimuli [4,10,11,15,16,17]. These receptors are tuned to sense specific stimulus features which focus on deciphering distinct pieces of contact/object properties. The dominant stimulus features are normal pressure (Merkel cell receptors [10,11,15]), horizontal motions and slip (Meissner corpuscle receptors [10,11,15]), vibrations (Pacinian corpuscle receptors [10,11]), stretch (Ruffini endings [10,11]) and proximity/approach (tylotrich-hair receptors [10,11]). The receptors’ stimulus feature selectivity is influenced by the location of skin receptors in different dermal layers, by the deployment pattern, and by mechanical filter mechanisms. The receptors’ selectivity samples complex multi-modal stimuli to simple distinct uni-modal stimulus features allowing for the encoding of complex tactile information and selective attention. Peripheral axons connect these tactile receptors to the nerve cell bodies in the dorsal root ganglion next to the spinal cord [4,10,18,19,20], forming together the tactile part of the peripheral somatosensory system. Throughout all its parts, the somatosensory system maintains the somatotopic order of the conveyed and relayed information, that is, the relative spatial structure of its receptors is reflected in the order of its nerve fibers and nerve cells [4,10]. Ascending along the spinal cord towards the somatosensory cortex, the somatotopically ordered information of different body parts is assembled to a comprehensive sensory representation of the whole body, the homunculus [1,4,10,20]. These two dominant principles of biology for realizing the complex sense of touch, namely decomposing complex contact features of physical interactions to simple uni-modal stimulus features, and maintaining and assembling relative spatial information, naturally impacted the development of large-area e-skin systems [8,21,22].

Research in the last decade focused on different scalable multi-modal e-skin systems suited for large-area applications [23,24,25,26,27,28,29]. Some of these works [25,28,29] led to viable solutions targeting the challenges of distributed e-skin systems. However, these works have not yet sufficiently addressed the major challenge to handle a large amount of tactile information that an upscaling of e-skin would produce to cover large areas. The lack of a systematic approach for solving this remaining challenge explains why LASSs are not yet as available and widely utilized as other sensing systems such as auditory or visual.

Neuromorphic systems that employ event-driven information handling to increase the processing efficiency and to reduce the latency of systems have already been introduced more than two decades ago in the works of References [30,31]. Over the years, different implementations and event representations emerged optimizing the event-driven approach for different applications. The most notable approaches towards Event-Driven Systems (EDSs) are the neuromorphic Address-Event-Representation (AER) [30,31], the Send-on-Delta Principle (SoDP) [32,33,34], and more recently, the Asynchronous Encoded Skin (ACES) [35]. Some of these EDSs have been used in applications with e-skin [35,36,37], supporting their effectiveness and efficiency. However, none of these event-driven e-skin approaches fully consider the implications and challenges of effective deployment over large areas and its eventual system integration. This work does not target to introduce yet another principle for representing and handling events, it rather consolidates the findings of these previous works, homogenizes their underlying theory, and assesses their applicability in LASSs. Besides the introduction of the realization principles that we propose for flexible, feasible, and efficient event-driven LASSs, this work also aims to foster the exchange of perspectives and requirements of the different research fields, especially within the context of event-driven information handling and large-area tactile sensing.

Our initial works [38,39,40] demonstrated the effectiveness of the event-driven approach for handling the large amount of tactile information of LASSs in various experimental setups. Now, this work intends to provide a solid foundation for realizing and understanding event-driven LASSs in general with an emphasis on three points. First, *flexibility/deployability*, that is, neuromorphic hardware may be utilized but is not strictly required and the system can be adjusted with a reasonable amount of effort. Second, *feasibility/effectiveness*, that is, the presented principles are implementable, scale, and enable real-world applications. Third, *efficiency*, that is, the event-driven system outperforms its clock-driven counterpart with respect to network traffic and CPU load. A summary of our implementations and the experimental results of our work delivers the impacts and validation of the presented design and realization principles.

### Outline

Section 2 presents the challenges of LASSs, surveys existing solutions and design concepts, and introduces the remaining challenges. Section 3 presents the concept of efficient event-driven information handling, and analyzes existing approaches for EDSs and their applicability in LASSs. Section 4 presents the designs for realizing event-driven sensing in e-skin systems, including the design of event generators and their correct parameterization. Section 5 presents the designs for realizing event-driven information handling for LASSs in standard computing systems. Section 6 summarizes and connects the designs to the challenges they tackle, and how their realization impact the efficiency and effectiveness in our e-skin implementation. Finally, we conclude in Section 7.

## 2. Large-Area Skin Systems—*Challenges and Organization*

This section summarizes and highlights the specific challenges encountered when designing and realizing LASSs. The presentation goes beyond summarizing the results of previous studies [8,41]. A consolidated list of challenges constitute the base for assessing the applicability of event-driven approaches in LASSs.

### 2.1. Challenges of Large-Area Skin

In the following, we introduce and explain the most notable challenges in realizing LASSs. Each of the challenges will be labeled from C1 to C6.

#### 2.1.1. Reliability and Robustness (C-1)

Mechanical interactions stress and wear on a physical system. The standard method to protect these systems against those effects are caging and protecting fragile parts while keeping the spatial extension as compact as possible. Ensuring reliability and providing robustness is a non-trivial task in LASSs, since theses systems are highly distributed and endure physical contacts.

#### 2.1.2. Deployability (C-2) and Wiring (C-3)

Distributing thousands of tactile sensors throughout the body and wiring them to supply the sensors with power and low latency connections is challenging. Deploying and wiring the sense of touch strictly requires a systematic approach since the individual and unorganized placement and connection of a large amount of discrete sensors is totally infeasible.

#### 2.1.3. Localization (C-4)

The coupling between sensing and *locating a stimulus*, that is, determining of its pose (position and orientation) with respect to a point of reference, is of paramount importance in vision as well as in touch. Body surfaces covered with skin are three dimensional and not completely rigid. Additionally, an actuated body changes the relative positions between its body parts and thus the positions between the skin sensors. Consequently, the identification of skin sensor locations with respect to a body part exhibits a complex challenge, even if the sensors have been systematically deployed. Manually assigning the poses of thousands of sensors can become infeasible and a LASS could benefit from automated methods to acquire the location of its sensors.

#### 2.1.4. Low-Latency (C-5) and Efficiency (C-6)

Sensing systems need to provide and represent information with *low-latency* and enable *efficient information handling* in order to realize fast system responses. Implementing low-latency and efficient information handling turns more challenging with an increasing number of sensors and is particularly challenging in LASSs. In most applications, both sensing systems have to handle a huge amount of information within short periods. While high speed connections between sensors and information handling systems are feasible in concentrated systems, for example, cameras, they are hard to realize in distributed systems with many connections over long varying distances. Long distance connections and high bandwidths increase the influences of noise, crosstalk, reflection, and distortion resulting in the loss of signals, and failures in the power distribution, that is, signal and power integrity are harder to maintain when distance and bandwidth increase. Consequently, realizing low-latency connections between the distributed tactile sensors and handling a large amount of tactile information (e.g., 1260 skin cells @ 250 Hz, clock-driven: 315,000 packets/s, 29 MB/s) [40] are both demanding challenges in LASSs.

### 2.2. Key Principles for Distributed E-Skin

The tackling of the previously presented challenges requires a systematic approach since solutions targeting only one particular challenge might completely contradict or hinder solutions targeting the other challenges. Recent progress in realizing e-skin systems revealed that following the bio-inspired principles of *modularity* and *self-organization* within a system approach contributes towards solving and mitigating the first four challenges of LASSs [29,42]. The following sections briefly summarize the impacts of these two principles and their limits towards solving/mitigating the challenges of low-latency and efficiency.

#### 2.2.1. Modularity of Sensing Elements

Modularity breaks down a complex system into smaller less complicated and exchangeable modules [29,42]. The simpler a module, the lower the number of points-of-failure. Modules allow the containment of errors and the introduction of redundancy, enhancing the robustness of systems. Dividing a system into exchangeable and modular parts also contributes to the maintainability, flexibility, and deployability of a system. A modular system is modifiable and customizable, thus optimizable for specific deployment scenarios. Structuring modules into hierarchical entities (e.g., skin patches) further simplifies the deployment of a large number of modules. Rather than deploying each component of a module (e.g., a sensor), or each module of a group of modules, a set of modules can be deployed at once. Modularity significantly contributes to the deployability of a system.

#### 2.2.2. Self-Organization of Communication and Structure

A system that is not only modular but additionally self-organizing significantly contributes to the feasibility of large systems with thousands of modules. Self-organizing modules can form networks with short connections between neighbors rather than requiring long point-to-point connections between each module and a hub. A meshed network of modules simplifies the wiring challenge while it introduces at the same time connection redundancy that enhances the robustness of the system [29]. Self-organizing modules are also instrumental to automatically determine the structure of the deployed e-skin and the spatial distribution of its sensors, providing a solution for the infeasible task to manually determine the poses of thousands of tactile sensors [43]. Dynamic networking routing can further enhance the robustness of a self-organizing network of modules. This dynamic routing enables the automatic on-line reshaping of communication trees in meshed networks to handle broken connections or unbalanced communication loads without the need to restart the system [44].

#### 2.2.3. Limitations of Modularity and Self-Organization

Systems of self-organizing modules rely on modules with local processing capabilities. Besides realizing self-organization, these local processing capabilities can be exploited to filter [29] or fuse information [45] in the modules, reducing the computational load at the higher processing layers. However, the concepts of modularity and self-organization alone cannot directly contribute to solve the challenges of efficiently handling a large amount of tactile information with low latency.

## 3. Efficient Event-Driven Information Handling for Large-Area Skin Systems

This section introduces the concepts of efficient event-driven information handling and examines EDSs regarding their applicability in LASSs. Complex systems, artificial or biological, combine sensation, communication, processing, and actuation to achieve desired system behaviors; they need to *handle information*. Handling information not only refers to processing information, it rather addresses the complete information flow in a perception-action loop [46] or a system control loop, that is, acquiring, transmitting, processing, and acting on information [47]. In this sense, achieving the desired system behavior fundamentally depends on the fast, efficient, and loss-less representation, processing, and exchange of information.

The representation and conveyance of information in biology follows schemes quite different to the principles utilized in technical systems. The representation in biology could neither be described as analog nor digital. Biology uses binary action potentials, often also termed spikes or events, to represent and convey information between neurons [47]. These action potentials alone convey only a very limited amount of information. Action potentials in nerve fibers are either present or not, they do not convey any additional information, for example, in their shape and so forth. Information in biology is encoded in the spatio-temporal activity patterns in massively parallel nerve bundles or populations of neurons [11,17]. These neural codes employ a set of different information representation principles, which are: (1) type code, (2) spatial code, (3) rate code, (4) temporal code, and (5) latency code [11,17,48]. All these principles show that biology uses structure and time (spatio-temporal features) to encode and represent information. Although there has been a long debate if biology employs rate coding or temporal coding [48,49], the nervous system employs both. All the previously discussed information representation principles can be observed in the somatosensory system, and thus also in the sense-of-touch.

The limitations of traditional approaches, especially in applications which need to handle a large amount of information within short periods, triggered the development of spike-based bio-inspired and neuromorphic systems to mirror the incredibly high information handling efficiency of biological systems. These neuromorphic systems employ spike-based information representation principles in sensing, communication, and processing [30,31,36,37,50,51,52,53,54,55,56,57,58,59,60,61,62,63,64,65,66,67,68] and report major improvements in efficiency and speed, that is, the systems require less power, and handle information with higher temporal resolution and less latency. Spike-based neuromorphic systems exploit all neural codes found in biology and are realized on customized hardware with special asynchronous circuits, optimized for spike-based signals and processing, and mimicking neural computation principles. In this work, we focus less on mimicking biology in all its aspects of information representation and computation principles, we rather focus only on two basic principles which we belief contribute significantly to improve the efficiency and speed of systems, and connect well to traditional information theory. First, rather than concentrating on different neural codes to convey information with spikes, we summarize and simplify the concept to the principle of *event-driven information handling*, that is, only novel information, the events, drive the whole system. This simplified principle can still be considered as biologically inspired. Even employing different coding principles, the activity in populations of neurons is usually triggered (or inhibited) by the arrival of stimuli. This is particularly true for the afferents of sensory neurons. Their peripheral axons only generate action potentials when their receptors register stimuli and are otherwise silent, regardless to the neural code they utilize for conveying information [11,47]. Following this line of thought, we furthermore neglect rate coding and equalize event-driven systems with novelty-driven systems. We solely concentrate on the sparsity aspect of spiking neural networks since we target to exploit its *temporal redundancy reduction* and *saliency* enhancement capabilities. Temporal redundancy reduction and saliency positively contribute to system efficiency since less information has to be handled.

The biological information representation and handling principles have inspired the development of bio-inspired technical systems which might prove feasible for tackling the challenge of efficiently handling information in LASSs. In the following sections, we first formally describe the characteristics of Clock-Driven Systems (CDSs) (Section 3.1) and Event-Driven Systems (EDSs) (Section 3.2) to clarify terminology and definitions, and to eventually provide a homogenized presentation which simplifies the comparison of both systems. Then, we discuss the limits of clock-driven systems (CDSs) in Section 3.3. Afterward, we formalize and homogenize the common novelty-based event generation principle found in neuromorphic spike-driven systems and in systems that implement the time-discrete Send-on-Delta Principle (SoDP), in Section 3.4. Event generation principles do not differ between different implementations of event-driven systems, but event representations do. Therefore, Section 3.5 surveys the most prominent approaches towards representing events in EDSs. Based on this survey, we proceed with a comparative study and select the most applicable event representation for LASSs in Section 3.6.

### 3.1. Clock-Driven Systems (CDS)

Time-discrete systems follow the Nyquist-Shannon sampling theorem that defines constraints for the lossless conversion of time-continuous signals to time-discrete signals. The Nyquist-Shannon sampling theorem [69] states that any bandwidth limited time-continuous signal x(t) with t∈R can be represented by a time-discrete signal $x(t_{k})$ with $t_{k}=k\;T_{s}$ and k∈Z as long as the sampling frequency $f_{s}$ surpasses the bandwidth *B* of x(t) by at least a factor of two:

(1) $$\begin{array}{cc} f_{s} & >2\;B. \end{array}$$

Consequently, time-discrete systems ensure that a clock with at least a frequency of $f_{s}$ drives the information handling such that the constraint of the Nyquist-Shannon sampling theorem is fulfilled at all times and information loss is zero through all stages of the system. These systems are termed Clock-Driven Systems (CDS). Standard computing systems are CDSs and usually either implement the von Neumann [70] or the Harvard architecture [71].

### 3.2. Event-Driven Systems (EDS)

At the beginning of Section 3, we outlined that many works introduced spike-based neuromorphic systems mimicking the information representation and neural computation principles found in nature [30,31,36,37,50,51,52,53,54,55,56,57,58,59,60,61,62,63,64,65,66,67,68]. Since these systems are spike-driven, it is valid to describe them as event-driven systems (EDSs). In this work, however, we focus only on one particular kind of EDSs, that is, novelty-driven systems [51,52,59,72]. In contrast to the general group of spike-based neuromorphic systems, which use all neural coding principles, novelty-driven systems focus on sparse information representation, that is, neural time coding, and follow the idea that only novel information should drive a system. For simplicity, in this work whenever we refer to EDSs, we refer to the subgroup of novelty-driven systems (NDSs) and solely focus on their dominant characteristics.

Novelty-driven systems relate very well to one core statement in information theory [69]. In many applications, following the guideline of the Nyquist-Shannon sampling theorem, that is, realizing CDSs, results in a stream of samples containing a huge amount of uncontrolled redundant information. Temporally redundant information is especially apparent when the system continuously samples the same value. Systems can avoid temporal redundancy when they only handle novel information, that is, when they are only active when sensors register activity. Shannon’s information entropy and his source coding theorem formally describes the information rate of information sources, and thus how much information, or respectively redundancy, a signal contains [69]. The information entropy H(X) evaluates the probabilities $P(x_{i})$ of symbols $x_{i}$, that encode the information produced by the information source x(t)

(2) $$\begin{array}{cc} H(X) & =-\sum_{i=1}^{n}P\left(x_{i}\right)\log_{2}P\left(x_{i}\right) \end{array}$$

and is measured in bits. Thus, if an information source x(t) continuously emits the same signal level, then all probabilities $P(x_{i})$ besides one are zero and the information entropy H(X) is zero. Consequently, the signal does not contain information and repeatedly sampling it just produces uncontrolled redundancy and wastes resources. On the other hand, if x(t) is constantly changing, then the probabilities $P(x_{i})$ are more distributed and the information entropy H(X) is well beyond zero. Thus, sensors that register substantial changes in x(t) produce a considerable amount of information.

In summary, novelty corresponds to activity and is expressed by changes. Thus, systems driven by events, where events solely express novel information, avoid uncontrolled temporal redundancy throughout all stages, and gain efficiency simply due to the fact that information is represented more sparsely and less information has to be gathered, transmitted, and processed.

### 3.3. Handling Large Amount of Information

Up to date, most technical systems are clock-driven and handle information strictly following the Nyquist-Shannon sampling theorem. CDSs have been successfully applied in many different applications such as the high-speed precision motor control in hard drives [73], control of robot arms [74], and many more. These systems not only prove that CDS provide viable solutions, they usually achieve excellent performance.

Nevertheless, CDSs that require fast reaction times in real-time applications depend on high-bandwidth information resulting in high sample rates. High sample rates only marginally impact systems that handle a limit amount of information of a few sensors, for example, the position control of electrical motors. However, when systems have to handle a large amount of information with high sample rates, their realization may become challenging or even infeasible [8,75,76,77,78,79]. The challenge of handling a large amount of information with high sample rates emerges in systems that need to react fast to visual, auditory, or tactile (cutaneous) information. All these senses employ a large number of sensors. To handle a large amount of information with high sample rates, CDSs have to employ very powerful transmission and computing systems with severe demands on power and space [75,76,79]. While power and space mainly cause monetary and environmental disadvantages in stationary systems, both factors tremendously impact systems in mobile applications [32,33,76,77,79].

### 3.4. Event Generation/Event-Driven Sensing

EDSs, or more specifically NDSs, gain their efficiency by coupling their activity to the information rate of information sources, see Section 3.2. By this means, EDSs succeed in canceling the temporal redundancy observed in the sampled information of CDSs. Thus EDSs are more efficient than CDSs. On average, EDSs need to process less information, thus induce less latency, and consume less power.

This section focuses on novelty detection, that is, on the procedure and formalisms to decide if signals, for example, of sensors, provide valuable information. Since the amount of information a signal provides correlates with the amount of its changes, see Section 3.2, a novelty detector is basically a change detector that triggers activity, or respectively the generation of events. NDSs and change detectors have been introduced in two different research fields, in the field of energy efficient sensing, signal processing and control from the information and control theory point of view [32,33,34], and in the field of neuromorphic systems from the mimicking neural codes and neural computing principles point of view [30,31,36,50,51,52,55,61].

Neuromorphic systems realize the change-detectors for their event generators in analog circuits [51,52,55,59,80]. These circuits directly detect changes in analog and thus in time- and range-continuous signals x(t) and convert them to events $e_{i}$. The change detectors of the other field [32,33,34] are realized in *compound architectures* [33], that is, the information source, for example, a sensor, is first sampled with a sample rate of $f_{s}=1/T_{s}$ at time instances $t_{k}=k\;T_{s}$, k∈Z. Then, a digital, time-discrete change detector transforms the samples $x(t_{k})$ to events.

Analog change detectors monitor a signal x(t) and track the change of this signal until the accumulated change exceeds a predefined threshold δ. Therefore, the change detector integrates the derivative $\dot{x}\left(t\right)$ of the input signal x(t) until the integration reaches or passes the threshold δ

(3) $$\begin{array}{cccccc} \delta & \leq |\int_{t_{i-1}}^{t}\dot{x}\left(t\right)\;dt| & \; & ⟺ & \; & e_{i}\;at\;t=t_{i} \end{array}$$

at time instance $t=t_{i}$. At this time instance $t_{i}$, the information of the monitored signal is classified as novel and the change detector triggers the creation of an event $e_{i}$ that contains this novel information. The more precise the occurrence time $t_{i}$ of the event matches with the time instance of the actual signal change, the higher the *temporal precision* of the event generator is. Thus, any non-deterministic or non-constant delay between the actual signal change and the occurrence of the event reduces the temporal precision. Considering the properties of Riemann integrals, we can derive a relationship between an integral of a signal f(t) and its average $\bar{f(t)}$ in an interval t∈[a,b]:

(4) $$\begin{array}{cc} {\left[\bar{f(t)}\right]}_{a}^{b} & =\frac{1}{b-a}\int_{a}^{b}f\left(t\right)\;dt. \end{array}$$

Combining Equations (3) and (4) to

(5) $$\begin{array}{cc} \delta & \leq |{\left[\bar{\dot{x}\left(t\right)}\right]}_{t_{i-1}}^{t}|\cdot \left(t-t_{i-1}\right). \end{array}$$

indeed shows that the change detector evaluates the accumulated average change of the input signal since the occurrence of last event $e_{i-1}$ at time instance $t_{i-1}$, simplifying Equation (3) to:

(6) $$\begin{array}{cccccc} \delta & \leq |x\left(t\right)-x\left(t_{i-1}\right)| & \; & ⟺ & \; & e_{i}\;\mathrm{at}\;t=t_{i} \end{array}$$

Thus, a change detector in fact triggers the creation of events whenever the difference between the signal $x(t_{i-1})$ that caused the last event $e_{i-1}$ and the currently monitored input x(t) exceeds a specified limit δ. Because the analog change detector can trigger events at any time, its temporal resolution is only limited by the bandwidth of the analog circuits and can theoretically achieve an almost infinite equivalent sampling rate.

Digital change detectors monitor the samples $x(t_{k})$ with $t_{k}=k\;T_{s}$, k∈Z of a signal x(t). The underlying principle for detecting changes is similar to the analog change detector. A digital change detector also integrates the derivative $x(t_{k})$ of the sampled input signal $x(t_{k})$ but the integration process is digital and clock-driven. The integration continues until it passes the threshold δ

(7) $$\begin{array}{cccccc} \delta & \leq |\int_{t_{K_{i-1}}}^{t_{k}}\dot{x}\left(t\right)\;dt| & \; & ⟺ & \; & e_{i}\;\mathrm{at}\;k=K_{i} \end{array}$$

at time instance $t_{k}=t_{K_{i}}$ or respectively at the sample $k=K_{i}$. At time instance $t_{K_{i}}$, the sample is classified as novel and the change detector triggers the creation of an event $e_{i}$. Similarly to Equation (4), we can derive a time-discrete relationship between the average and the integral of a signal

(8) $$\begin{array}{cc} {\left[\bar{f(t)}\right]}_{t_{K_{1}}}^{t_{K_{2}}} & =\frac{1}{K_{2}-K_{1}}\sum_{l=K_{1}}^{K_{2}}f\left(t_{l}\right), \end{array}$$

which combined with Equation (7) leads to

(9) $$\begin{array}{cc} \delta & \leq |{\left[\bar{\dot{x}\left(t\right)}\right]}_{t_{K_{i-1}}}^{t_{k}}|\cdot \left(t_{k}-t_{K_{i-1}}\right) \end{array}$$

and simplifies to:

(10) $$\begin{array}{cccccc} \delta & \leq |x\left(t_{k}\right)-x\left(t_{K_{i-1}}\right)| & \; & ⟺ & \; & e_{i}\;\mathrm{at}\;k=K_{i}. \end{array}$$

Thus, the digital time-discrete change detector has to remember the signal sample $x(t_{K_{i-1}})$ of the previous event $e_{i-1}$ and compare it to the current sample $x(t_{k})$. Then, when the absolute difference between these two samples exceeds the threshold, the change detector triggers the creation of the event $e_{i}$ and updates the memory with the current sample. In comparison, digital change detectors naturally exhibit a lower temporal resolution than their analog counter parts and consume more power since the monitoring is clock-driven. The temporal resolution of digital change detectors is limited by the sampling rate of the digital system.

### 3.5. Event Representation / Information Encoding

This section focuses on the different event representations and transmission techniques found in the most notable approaches towards realizing EDSs. The surveyed representations include the most common representation applied in neuromorphic systems [30,31], the representation applied in the event-driven sensing and control community [32,33,34], and one recently proposed representation technique developed for e-skin systems [35]. Event representation is tightly coupled with encoding information in events, since these events eventually carry information in EDSs. This subsequent study will discuss the applicability of the different approaches towards realizing an effective novelty-driven event handling system for LASSs.

#### 3.5.1. Address Event Representation (AER)

The Address Event Representation (AER) [30,31] is one of the first bio-inspired systems that has been developed for representing and conveying events in technical systems. Originally, AER has been developed for the communication between spiking artificial neurons in VLSI ICs (Very Large Scale Integrated Circuits) [30,31] and rigorously takes advantage of high-speed digital asynchronous parallel bus systems that are readily available on such devices. AER realizes event-driven point-to-point connections between event generators and consumers. But instead of encoding the information source by individually wiring each event generator to an event consumer, as nature does, the AER employs addresses to identify sources and time-multiplexes these addresses onto a common asynchronous parallel bus. A valid address on this bus represents an event and this address identifies the event generator of that event. The AER exploits the superior communication speed of technical systems per wire in integrated systems (>100 MBit/s) in comparison to nerve fibers (≈1 kBit/s) to reduce the number of wires and still achieve a comparably high temporal resolution. Besides the address bus, the AER employs a request, and an acknowledge line to realize a self-timed bus arbitration mechanism that avoids any clock resynchronization. AER represents events through addresses. To convey information, AER event generators can employ encoding principles that are similar to neural codes. The AER can encode the type of events in additional address lines such that an AER event generator can create change events, events that indicate an increase or decrease of the observed signal (up and down events), respectively [51,52]. To encode absolute values instead of increments, AER event generators can employ low and high events where the time between these two events represents the encoded value [55]. More recent research introduced serial AER [81] to reduce the wiring complexity in more distributed EDSs [37]. Serial AER packs the event address into a datagram on a serial bus, which reduces the number of wires at the cost of reducing the temporal resolution. The AER is an established bio-inspired protocol for representing and transferring events and could successfully demonstrate its use in auditory [50], visual [51,52], and force [36] sensing applications and in event-driven processing hardware such as SpiNNacker [57], TrueNorth (IBM) [58], BrainScaleS [62], ROLLS [63], DYNAP [66], Loihi (Intel) [67], and BrainDrop [68].

#### 3.5.2. Send-On-Delta Principle (SoDP)

The Send-on-Delta-Principle (SoDP) [32,33,34] is a hybrid system which exploits standard digital hardware to realize EDSs. The SoDP has been first proposed for efficiently reducing the number of transmissions in wireless, battery powered, and widely distributed sensors networks [32,33]. In these application scenarios, the reduction of the number of transmissions is essential to increase the life time of the distributed sensors. SoDP systems employ time-discrete digital change detectors, that can even be implemented in software, to trigger the creation of events. The SoDP represents events by packets (event packets) that are transported in asynchronous arbitrated networks. An event packet usually contains the ID of its information source and the absolute value of the signal at the creation time of the packet. Similar to the AER, the presence of an event packet signifies the availability of novel information and drives the information handling of the system. Since the SoDP not only conveys the information source but also the absolute magnitude of the signal, the bit rate of SoDP events is higher than the bit rate of AER events. Thus, the temporal resolution of SoDP systems is lower than that of AER systems when both system employ the same transmission rate. However, decoding the information of SoDP events is by far less complex than for AER events whenever an application enforces clock-driven information, for example, in low-level control of closed hardware/software systems, such as in robots. Furthermore, SoDP systems do not require specialized hardware and can be realized with off-the-shelf sensors and well established information transport layers. Actually, CDSs which possess the flexibility to modify their information handling procedures and can employ asynchronous transmission and processing capabilities can be turned into EDSs without the need of any hardware modifications. The SoDP provides great flexibility and availability for realizing low cost and scalable event-driven applications. However, the hardware of SoDP systems is clock-driven such that SoDP cannot reach the temporal precision and energy efficiency of systems that employ event-driven neuromorphic hardware.

#### 3.5.3. Asynchronous Encoded Skin (ACES)

The Asynchronous Encoded Skin (ACES) [35] is an event-driven hardware system that has been recently proposed to realize a neuro-inspired artificial nervous system. The ACES implements a many-to-one protocol for transmitting and representing events. Rather than time-multiplexing events to a common transportation medium such as in the AER or SoDP, the ACES fuses events as pulse signatures onto one single common wire. A pulse signature is a sequence of pulses within a constant time window, where the relative timing of the pulses encode the signature. Similar to the addresses in AER and the IDs in SoDP, the pulse signature identifies the information source and represents the event. Interestingly, the ACES manages to fuse these pulse signatures on one single wire without applying time-multiplexing or requiring an arbitration method. The ACES superimposes all pulse signatures by applying a logical OR operation on the pulses. In order to minimize the probability that pulse signatures cannot be separated, the set of pulse signatures has to have minimal auto-correlation and cross-correlation. Theoretically, ACES could support up to 138,000 information sources per wire, when a pulse signature has a time window of 1 ms, consists of 10 pulses, and each pulse lasts for 100 ns. In such a setup, ACES events have a latency of at least 1 ms when employing up and down events, or respectively a latency of at least 2 ms when employing low and high events for time-coding absolute values. However, the temporal precision of ACES is extremely high (in the range of the pulse length) since no arbitration mechanisms impair the temporal precision with non-deterministic uncertainties in delay which correlate with the utilization of a shared communication medium. Additionally, since the ACES event transmission is arbitration-less, connection redundancy could be introduced by adding wires as long as the propagation speed and the reflection of high speed connections do not degrade the transmission quality. While the hardware for encoding and representing events in ACES has a low complexity, acquiring a set of pulse signatures is more demanding and the demerging of ACES events is very complex. An ACES event demerger has to repeatedly correlate the currently observed pulse pattern of superimposed events with all pulse signatures of the set. Therefore, the ACES event demerger has to keep a history of received pulses which matches the length of a pulse signature. To preserve the temporal information of the events, the demerger has to perform this correlation continuously for each potential event in parallel within the time length of a pulse. For the example numbers mentioned earlier, the demerger would at least have to perform continuously 138,000 correlations with a bit length of 10,000 bits (assuming a pulse can be represented by one bit) within 100 ns. The ACES event decoder is clearly not event-driven since the decoding has to be driven by the pulse time, and the information in the superimposed pulse stream is not salient. Nevertheless, the demerged events can drive the information handling in subsequent stages.

### 3.6. A Comparative Study of Effective Event Representation for Large-Area Skin Systems

Section 3.5 introduced and described existing event representations and their realization in EDSs. These realizations have been successfully validated and proved their efficiency in various applications. To assess which EDS approach suits best for LASSs, we examine and discuss their performance within the relevant properties, see Table 1. All properties are assessed considering the challenges of LASSs summarized in Section 2.1.

The predominant factor for proposing EDSs for LASSs is their information handling efficiency. Next to efficiency and latency, an effective EDS for LASSs has to consider also robustness, deployability, wiring complexity, and sensor poses. To tackle these challenges an EDS should support the principles of modularity and self-organization.

Table 1 summarizes the most important properties of EDSs. It also includes the properties of nerve bundles and CDSs to enable comparisons with the biological reference and with the state-of-the-art approach in technical systems. In the following assessment, we focus on the properties’ most important implications for LASSs before selecting the most suitable EDS.

#### 3.6.1. Connection, Bandwidth, and Arbitration

Standard AER employs parallel asynchronous buses with a handshaking mechanism. This bus can provide very high bandwidths but is unidirectional and utilizes many wires. Therefore serial-AER has been introduced to reduce the wire count at the cost of a slight reduction in bandwidth. The AER time-multiplexes events on a common bus and thus has to employ very complex arbitration mechanisms to ensure fair sharing and to optimize temporal precision. The arbitration latency depends on the bus utilization which is non-deterministic and correlates with the global information rate. The complex handshaking mechanisms require special hardware and are rather inflexible and hard to change.

The communication protocol of ACES has been specifically designed to reduce the complexity of merging the events of multiple information sources to a common transport medium. ACES has clear advantages over AER with respect to wire count and flexibility. ACES exploits the uniqueness of its events to completely avoid any arbitration. Avoiding arbitration, ACES achieves a lower circuit complexity, and a higher temporal precision than AER. Furthermore, information sources and wires can be added/removed in ACES without the need to consider and adjust a complex arbitration system. This ability greatly increases the robustness and flexibility of ACES in comparison to AER. However, the bandwidth of ACES is several orders of magnitude lower than in AER.

In contrast to AER and ACES, SoDP does not rely on a specifically designed transport medium for conveying SoDP events. Any protocol and hardware that asynchronously conveys packets is suitable for SoDP. The hardware independency allows for the extreme flexibility, robustness, and the rapid implementation of SoDP-based EDSs. Nevertheless, the SoDP has to time-multiplex and arbitrate events to share a common communication medium. But in contrast to AER, the arbitration is much more flexible, less complex and can be achieved by standard network protocols. Naturally, the temporal precision of SoDP is lower than in AER and ACES, since SoDP events require more bits and thus occupy a shared bus for a longer time. The higher bit count per event in SoDP reduces the overall communication bandwidth below the one of AER but still well beyond ACES.

#### 3.6.2. Representation and Encoding of Events

In AER and ACES the events solely encode the source and the type of information while the information itself is encoded in the timing/occurrence pattern of the events. As a result, an event can be represented by few bits and only demands a tiny part of the transport capacity on a bus rendering these systems highly efficient. Nevertheless, the event conveyance system has to exert a high temporal precision since the information is encoded in the timing of events. To achieve such high temporal precision, both systems rely on specialized hardware.

On the other hand, SoDP does not rely on neural codes and does not only encode the source and type of information into and event but also the information itself, that is, an absolute value. Consequently, the temporal precision is less critical than in AER and ACES but still important. The occurrence time of a SoDP event still encodes the occurrence time of the information. The downside of SoDP events is that they require more bits and thus more communication bandwidth. While SoDP still constitute a major improvement towards tackling efficiency and low-latency, however, it cannot achieve the efficiency, latency and temporal precision of AER or ACES.

#### 3.6.3. Decoding of Events

Ideally, for handling information, EDSs should never experience the need to decode events to other representations such as samples of absolute values. Research in EDSs actually advances into that direction and progress in event-driven hardware and event-driven information handling develop to an emerging new research field [30,31,36,37,50,51,52,53,54,55,56,57,58,59,60,61,62,63,64,65,66,67,68] that will provide highly efficient information handling systems. However, many applications still, and for the foreseeable future will, rely on clock-driven information handling algorithms. Thus, to really profit from EDSs in applications, EDSs have to provide efficient event decoding mechanisms.

Decoding events in AER and ACES is complex and requires special hardware to convert events and their time encoded information to a format that can be processed by standard computer systems, that is, tagging high precision time stamps to AER events [51,52,55], or gray scale values [52,55]. While decoding AER events is complicated, demerging ACES events is really challenging. In AER and SoDP, the events on a common bus are salient and may directly drive the decoding or the handling of information in event-driven algorithms. However, ACES events are not salient and an event demerger has to constantly monitor and detect events in a massively parallel clock-driven process, even if the subsequent information handling stages are event-driven. The necessity of always decoding ACES events constitutes a negative impact on the information handling efficiency of ACES. In general, decoding AER or ACES events negatively impacts the efficiency.

Since SoDP events already encode absolute values, their decoding is simple. SoDP events are salient and their decoding can be event-driven. Thus, the decoding of SoDP events is more efficient than in AER or ACES.

#### 3.6.4. Effective Event Representation for Large-Area Skin Systems

Overall, the SoDP emerges as the most suitable EDS for tackling all the challenges towards realizing LASSs. While AER and ACES have advantages in achieving more efficient solutions than SoDP, they have also deficiencies. They require special hardware, complex decoding, and a complex setup, thus hinge on overall deployment. The clear advantage of SoDP lies in its great flexibility since it does not depend on specific hardware and can thus exploit standard hardware for the rapid realization of complex but yet efficient EDSs.

## 4. Realizing Event-Driven Sensing for Efficient Large-Area Skin

This section presents our designs for realizing event-driven sensing in e-skin systems and the expected efficiency gain in Section 4.1. The designs are further refined in Section 4.2 which discusses the event generation in the smart modules of e-skin systems. Furthermore, we introduce rules for the correct parameterization of event generators in Section 4.3.

### 4.1. Event-Driven Sensing in Large-Area Skin

Section 2 and Section 3 extensively discussed the challenges of LASSs and how these challenges may be tackled by employing modularity, self-organization, and bio-inspired event-driven information handling. We now focus on the actual realization of LASSs considering the collected insights and on the expected efficiency gain of the presented realization principles.

#### 4.1.1. Realizing Event-Driven Sensing in Large-Area Skin

**Figure 1:**
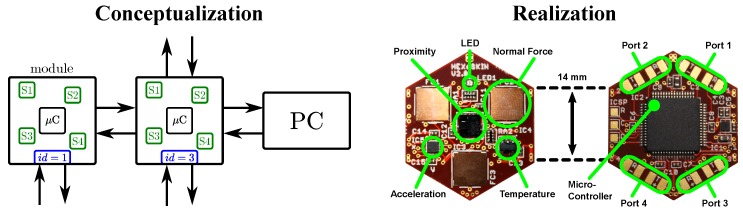
Conceptualization: Each sensor module/skin cell employs different sensors (e.g., S1, …, S4) and a microcontroller (μC) for local processing capabilities; the modules are inter-connected, form a network of modules, and provide a bidirectional communication path between each module and information handling systems, for example, a computer (PC); Realization: One skin cell of our e-skin system [29].

As discussed in Section 2, the best way to tackle most of the challenges of LASSs is a self-organizing and modular system. Therefore, a LASSs should group sensors in smart modules. We refer to a module as a *smart* module when the module provides local processing capabilities and a group of modules can distributedly organize themselves into a robust network of communicating skin cells, see Section 2.2 and Figure 1. These smart skin cells embed local processing capabilities (e.g., microcontrollers) that implement the self-organizing network capabilities in the network of skin cells. The realization of such a modular and self-organizing skin system then only lacks the information handling efficiency to effectively scale up to LASSs.

The efficiency of a modular and self-organizing skin system can be extensively improved by introducing event-driven sensing and by handling information through events. Section 3 concludes that SoDP suits best for this purpose. Realizing a event-driven modular and self-organizing skin system requires event-driven sensing at skin cell level, and event-driven communication between skin cells and the higher-level information handling. Therefore, we exploit the local processing capabilities of the skin cells and implement event generators following the principles discussed in Section 3.4.

The realization of the modular generation of events will be more extensively discussed in Section 4.2 before we focus on their sensing characteristics in Section 4.3. The modular event generators in the skin cells produce SoDP events that are packets in the skin cell communication network. Consequently, these packets can be handled by the self-organizing network like the packets of a CDSs, as long as the network allows asynchronous communication. Most state-of-the-art communication systems, such as RS232/UART and Ethernet/UDP fulfill this requirement. Realizing efficient event-driven handling of information in the higher layers will be elaborated in Section 5.

A clock-driven e-skin system that provides smart skin cells and a robust self-organizing communication network has been developed in our previous work [29]. Since this e-skin system provides all requirements, we use it to validate our designs towards realizing efficient event-driven LASSs, see Section 6.

#### 4.1.2. Event-Driven Sensing increases the Efficiency of Large-Area Skin

Event-driven sensing significantly reduces the networking and processing load in comparison to clock-driven sensing. A clock-driven e-skin constantly induces high information handling loads. These loads $L_{d}$ scale linearly with the sampling frequency $f_{s}$ and the number of sensors $n_{s}$:

(11) $$\begin{array}{cc} L_{d} & \propto f_{s}\;n_{s}. \end{array}$$

Contrarily, event-driven e-skins only require transmission bandwidth and processing power when they are active, that is, when they handle events. These loads depend on the event rate $f_{e}$

(12) $$\begin{array}{cc} L_{e} & \propto f_{e} \end{array}$$

and can be approximated by the number of activated sensors $n_{a}$ that register novel information

(13) $$\begin{array}{cc} L_{e} & \underset{∼}{\propto }n_{a} \end{array}$$

na or more concretely by the shape of the stimuli $x_{a}\left(t\right)$ these sensors register:

(14) $$\begin{array}{cc} L_{e} & \propto \sum_{a}^{n_{a}}\bar{|{\dot{x}}_{a}\left(t\right)|}. \end{array}$$

A perfect event-driven e-skin system would not create and handle events when its sensors do not register stimuli with novel information and its event rate $f_{e}$ would be zero. In the worst case, all sensors are activated and stimulated with maximum information rate. The event rate $f_{e}$ then reaches the cumulative sample rate of a CDS

(15) $$\begin{array}{cc} f_{e} & =f_{s}\;n_{s}, \end{array}$$

when utilizing discrete event generators in compound architectures, for example, SoDP. The event rate could even surpass that limit in systems employing neuromorphic event-driven sensors, since these sensors usually realize higher bandwidths than clock-driven sensors [51,52]. However, for e-skin systems, especially LASS, the worst case is very improbable. Touch stimuli are usually localized to spots on the body, and scenarios where the skin of the whole body is stimulated with a non-constant profile, that generates many events for a long time, are extremely rare. Even covering the LASS of a robot with a cloth and moving the cloth to create additional stimuli can by far not reach the excitation levels necessary to deteriorate the performance of the EDS to the performance of a CDS [40]. Consequently, the event rate

(16) $$\begin{array}{cc} f_{e} & \ll f_{s}\;n_{s} \end{array}$$

is on average much lower than the cumulative sample rate of CDSs. In summary, realizing event-driven information handling in LASSs will definitely improve their information handling efficiency and will thus contribute to render these system feasible to operate in real world applications.

### 4.2. Modular Event Generation for Large-Area Skin

**Figure 2:**
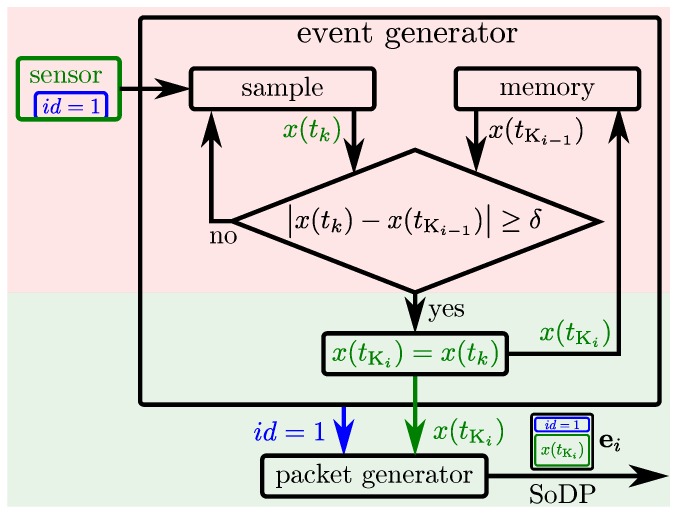
A networked module/skin cell with local processing capabilities, see Figure 1, can realize an event generator (EG) and an event packet generator (EPG) representing events in the SoDP; a sensor with an ID is sampled and its value $x(t_{k})$ is compared with the value $x(t_{K_{i-1}})$ of the previous event $e_{i-1}$; if the absolute difference is below δ, then the next sample is acquired, otherwise a new event $e_{i}$ is generated with a sensor value $x(t_{K_{i}})$; the EPG creates an event packet containing the ID of the sensor and the sensor value $x(t_{K_{i}})$; the red background indicates components that are strictly clock-driven, and the green background represents components that are event-driven.

Smart skin cells of a LASS provide local processing capabilities, see Section 4.1. The modular event generation at the sensor level exploits these capabilities to realize a time-discrete change detector, see Equation (10), for each of the skin cells’ sensors, see Figure 2. The skin cells continuously sample their sensors and compare the current sensor value $x(t_{k})$ with the sensor value $x(t_{K_{i-1}})$ of the previous event $e_{i-1}$. Whenever the absolute difference between the current sensor value and the value of the last event is larger than δ, then the change detector triggers the generation of a new event $e_{i}$. Events are represented in SoDP and an event packet generator (EPG) creates and transmits an event packet containing an ID to identify the sensor and the sensor value $x(t_{K_{i}})$ that caused the creation of the corresponding event. Skin cells that employ more than one sensor need to implement an event generator for each sensor *l* but can share the same packet generator.

**Figure 3:**
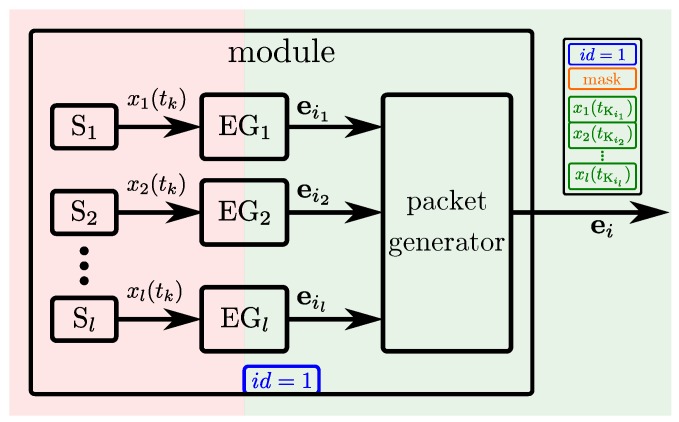
Skin cells/Modules employing several sensors need one event generator ${\mathrm{EG}}_{l}$ per sensor $S_{l}$; to reduce the overhead of sending an event packet for each sensor of a skin cell, the skin cell can stack events $e_{i_{l}}$ occurring at the same time $t_{K_{i_{1}}}=\cdots =t_{K_{i_{l}}}$ into one skin cell event packet $e_{i}$; the skin cell event packet contains the skin cell ID to identify the module, a mask to identify the sensor, and the sensor values $x_{l}\left(t_{K_{i_{l}}}\right)$ of the events $e_{i_{l}}$.

Since the spatial distribution of a skin cell’s sensors is close, and the temporal resolution of their sensors is limited by their sampling rate, the probability of exciting several sensors (of different modalities) at the same time is high. This probability leads to an optimal number of sensors per packet generator and has thus been analyzed for our skin system [38] to eventually reduce the overhead of event packets. Rather than each skin cell sending an event packet for each event of its sensors as depicted in Figure 2, each skin cell can pack all the events occurring at the same time in one common packet, the *skin cell event packet*, see Figure 3. Such a skin cell event packet thus contains all the events of a skin cell that occurred at the same time. The payload of these packets is depicted on the right side of Figure 3 and characterized by $e_{i}=\left(\mathrm{ID},\;\mathrm{mask},\;x_{1},\;\ldots ,\;x_{l}\right)$. Figure 3 depicts the event and packet generators within one skin cell. Skin cell event packets identify the skin cells by skin cell IDs, and the active sensors of a skin cell by a mask. Then, the sensor values $x_{l}\left(t_{K_{i_{l}}}\right)$ follow after the mask field.

The size of skin cell event packets thus changes with the number of sensor events that occur at the same time. While packets with dynamic sizes are usually supported by networks, some networks require a predefined static packet size. Then a good trade-off has to be determined between the overhead of sending several small packets when several events occur at the same time, and the overhead of sending partially empty packets when only one event occurs [38].

The design principle of modular event generation in the skin cells of an e-skin system has been implemented and validated in References [38,39]. Therefore, we exploited the local processing capabilities of the skin cells of our previously developed e-skin system [29,82]. More recently, we demonstrated the scalability and deployability of our e-skin system [83]. Transforming the clock-driven to an event-driven e-skin system proved to be essential in the large-area deployment as we evaluated in Reference [40].

### 4.3. Event-Driven Sensing: Parameterizing Event Generators

The modular event generators introduced in the previous Section 4.2 employ the discrete change detector of Equation (7) described in Section 3.4. This change detector uses a threshold value as tuning parameter which defines the amount of novelty that has to be gathered until a new event is generated. This threshold not only influences the boundary for novelty, it also impacts the event rate, the encoding error, the targeted sensitivity, and the susceptibility to noise. Therefore, this section concretely analyzes the correlation of these properties with the threshold, and for the first time derives guidelines for optimally tuning the threshold.

As outlined in Section 3.2, the event generators directly correlate the event rate $f_{e}$ with the information rate H(X) of the sensor:

(17) $$\begin{array}{cc} f_{e} & \propto H(X). \end{array}$$

In this context, the novelty threshold δ tunes the amount of change of x(t) required to justify that this change represents novel information. Thus, the threshold δ correlates with the smallest detectable change, and thus with the sensitivity of the event generator. A smaller δ results in a higher sensitivity and a smaller encoding error. In any case, the difference between the actual value x(t) and the last reported value $x(t_{K_{i-1}})$ of an event $e_{i-1}$ is at most δ. However, a higher sensitivity results in higher event rates that increase the overall system activity.

The upper bound of the event rate can be estimated by employing the event generation rule defined by Equation (10):

(18) $$\begin{array}{cc} \delta & \leq |x\left(t_{K_{i}}\right)-x\left(t_{K_{i-1}}\right)\left|=\right|\bar{{\dot{x}}_{i}}|\cdot T_{e,i}. \end{array}$$

This equation now describes the relation between the time $T_{e,i}=t_{K_{i}}-t_{K_{i-1}}$ between two consecutive events $e_{i-1}$ and $e_{i}$, the average slope of the input $|\bar{{\dot{x}}_{i}}|$ of these events, and the threshold δ. Then, the average event rate $\bar{f_{e}}$ can be estimated with [84]:

(19) $$\begin{array}{cc} \bar{f_{e}} & =\frac{n}{\sum_{i=1}^{n}T_{e,i}}\leq \frac{1}{\delta }\;\bar{|\dot{x}\left(t\right)|}. \end{array}$$

This estimation bases on two conservative assumptions. First, in discrete time systems, the time $T_{e,i}$ between events is limited by the sampling frequency, that is, if the average slope $|\bar{{\dot{x}}_{i}}|$ exceeds the limit defined by the sampling frequency $f_{s}$

(20) $$\begin{array}{cc} |\bar{{\dot{x}}_{i}}| & \geq \delta \;f_{s}, \end{array}$$

then $T_{e,i}$ is underestimated, thus $\bar{f_{e}}$ overestimated. Second, the average of the absolute slope $\bar{|\dot{x}\left(t\right)|}$ overestimates the sum of the absolute averages of slopes between events

(21) $$\begin{array}{cc} \bar{|\dot{x}\left(t\right)|} & \geq \sum_{i=1}^{n}\left|\bar{{\dot{x}}_{i}}\right|, \end{array}$$

whenever the slope $\dot{x}\left(t\right)$ changes its sign between events. Considering the result of Equation (19), it is obvious that for an identical input profile x(t) the mean event rate $\bar{f_{e}}$ inversely correlates with the threshold δ:

(22) $$\begin{array}{cc} \bar{f_{e}} & \propto 1/\delta . \end{array}$$

Any input signal x(t) composes of the actual stimulus s(t) superimposed with the background noise z(t):

(23) $$\begin{array}{cc} x(t) & =s(t)+z(t). \end{array}$$

Consequently, noise has to be considered when tuning δ. If z(t) is white noise with a normal distribution, an expected value $\mu_{N}$ of zero, and a variance of $\mathrm{Var}\left(Z\right)=\sigma_{N}^{2}$

(24) $$\begin{array}{cc} Z & ∼N(0,\sigma_{N}^{2}), \end{array}$$

then the standard deviation $\sigma_{N}$ of the noise can be used to guide the tuning of δ. In normal distributions, 68% of the values $z_{i}$ of the random variable *Z* lie within $\sigma_{N}$, 95% within $2\;\sigma_{N}$, and 99.7% within $3\;\sigma_{N}$. Consequently, anytime the signal s(t) stays constant, the average event rate $\bar{f_{e}}$ for x(t) generated by noise is

(25) $$\begin{array}{cccccccc} \bar{f_{e}} & =\left[1-\mathrm{erf}\left(0.5\;\sqrt{2}\right)\right]\;f_{s}\; & \; & \approx 0.31731\;f_{s}\; & \; & \mathrm{for} & \; & \delta =\sigma_{N}\\ \bar{f_{e}} & =\left[1-\mathrm{erf}\left(\sqrt{2}\right)\right]\;f_{s}\; & \; & \approx 0.045500\;f_{s}\; & \; & \mathrm{for} & \; & \delta =2\;\sigma_{N}\\ \bar{f_{e}} & =\left[1-\mathrm{erf}\left(\frac{3}{2}\;\sqrt{2}\right)\right]\;f_{s}\; & \; & \approx 0.0026998\;f_{s}\; & \; & \mathrm{for} & \; & \delta =3\;\sigma_{N}, \end{array}$$

where $f_{s}$ is the sampling frequency of the sensor or respectively the update rate of the event generator. The function erf(·) is the standard Gauss error function:

(26) $$\begin{array}{cc} \mathrm{erf}(x) & =\frac{2}{\sqrt{\pi }}\;\int_{0}^{x}e^{-t^{2}}\;dt. \end{array}$$

Intrinsically, the noise deviation $\sigma_{N}$ of the sensor has a major impact on the maximum sensitivity that can be chosen without generating too many events triggered by noise. Furthermore, we note that the number of events that are triggered by noise increases with higher sampling frequencies.

In summary, the following guidelines should be considered when tuning the threshold δ to minimize the event rate, to maximize the sensitivity, and to minimize the error:

1. when the maximum required encoding error is $\epsilon_{\max}$, then choose $\delta <\epsilon_{\max}$
2. when the required sensitivity needs to detect changes down to a minimal change $\Delta_{\min}$, then choose $\delta <\Delta_{\min}$
3. when the idle event rate, that is, the event rate for x(t)=const., or respectively the noise event rate has to be smaller than 1% of $f_{s}$, then choose $\delta >3\;\sigma_{N}$
4. when the overall mean event rate is too high, then
  (a) increase δ at the cost of reducing the sensitivity and increasing the encoding error
  (b) decrease $f_{s}$ at the cost of reducing the temporal precision
5. similar to the signal-to-noise ratio (SNR), the $\sigma_{N}$ of the noise source defines the performance limits of the event generator, and thus, has to be kept as small as possible.

These guidelines have been successfully applied in earlier experimental evaluations [38,40].

## 5. Realizing Event-Driven Information Handling for Large-Area Skin

This section presents our designs for realizing event-driven information handling for LASSs in standard computing systems without the need for special hardware. Section 5.1 details how the exploitation of the scheduling mechanisms of operating systems can lead to asynchronous computation on-demand, a key element of event-driven information handling. This examination results in designs for event-driven programs. The design of effective applications often requires interfaces between event-driven LASSs and clock-driven information consumers. Therefore, we discuss event decoders in Section 5.2 to bridge information between EDSs and CDSs.

### 5.1. Event-Driven Information Handling on Standard Computing Systems

**Figure 4:**
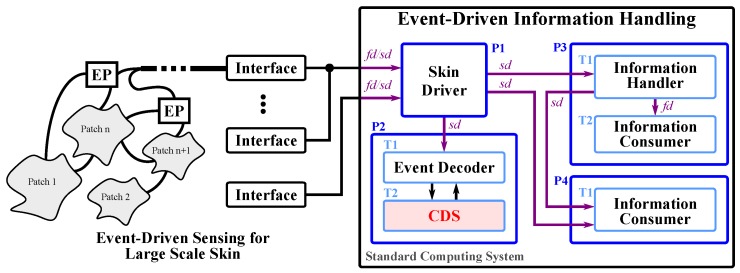
Event-driven information handlers exploiting the Signaled-Wakeup principle; *fd* are file descriptors, while *sd* are socket descriptors; Processes are denoted with P and threads with T; Process P2 contains a Clock-Driven Systems (CDS) and employs an event decoder to bridge from the event-driven to a clock-driven system; the endpoints (EP) between skin patches and interfaces refer to our realization of extended modularity in the interfaces; our new interface system differentiates the interfaces to main interfaces and interface satellites (EPs), see Reference [40].

An event-driven LASS not only relies on event-driven sensing in its deployed e-skin, see Section 4, it also heavily relies on the handling of its information at higher processing layers, see Figure 4. Specialized bio-inspired event-driven computing systems with massively parallel computing capabilities are emerging [57,58,62,63,66,67,68], but require special hardware. While these systems provide very good performance, the bridging to CDS, for example when connecting an event-driven system to a standard robot platform, requires complex algorithms and specialized hardware to decode events and provide information to the CDS. Therefore, we propose to employ SoDP on standard computing systems to provide a more flexible and practical realization of event-driven information handling for LASSs.

Standard computing systems utilize multi-core processors and multi-thread capable operating systems. These operating systems schedule concurrent tasks by pausing the execution of a task (preemption) and resuming the execution of another task. In this way, tasks can share computation time and, as long as the switching between tasks is faster than their required reaction time, these operating systems effectively realize processing that is virtually concurrent and asynchronous. Multi-core systems improve concurrency since several computations can physically take place at the same time. Operating systems handle the fair splitting of computation time (timeslices) between tasks (processes and threads) ensuring that tasks respond as requested. To increase the efficiency of scheduling, tasks can yield their computation time and ask the operating system to resume on the occurrence of a signal. We refer to this yielding and resuming on the occurrence of a signal [85] as *Signaled-Wakeup* principle (SWP), see Appendix A. This principle combined with multi-threaded programs allows us to realize an event-driven information handling system on standard computing systems. Our current implementation is realized on a Linux standard OS. The implementation could further profit from a real-time kernel with reduced latencies. Since the principles we exploit for realizing event-driven information handling on standard computing systems are available in many operating systems (e.g., Windows, Mac) our implementation is not strictly limited to Linux OS.

The resulting event-driven system consists of event generators and event consumers, see Figure 4. The design and realization of event generators has been discussed in Section 3.4. Event generators can be located inside and outside the computing system and we discuss their connection to signals in Section 5.1.1. We then discuss the design for event consumers in Section 5.1.2.

#### 5.1.1. Connecting Event Generators

To exploit the SWP for realizing event-driven information handling in standard computing systems, event generators have to trigger the signals (e.g., file/socket descriptors) of the operating system to wakeup and resume the computation of the newly arriving events in the event consumers.

Event generators and event consumers may reside on different hardware, for example, the event generators of a large-area event-driven skin reside in the skin cells of the e-skin system and thus outside the computing system, see Figure 4. These external SoDP event generators, see Section 4.2, create event packets that are immediately sent into the communication network and forwarded to the information handling system. Most operating systems connect the arrival of network packets directly to signals (*fd*/*sd*), see Figure 4, such that the events of an external event generator eventually trigger the signals of the operating system.

Event generators residing inside the computing system may connect to signals, and thus event consumers, in two manners. Threads that share the same process can use file descriptors (*fd*) to connect event generators, see process P3 in Figure 4. There, thread T1 (event generator) is connected to thread T2 (event consumer) via the file descriptor *fd*. Threads that are attributed to different processes can connect and share information through the local virtual network, see processes P3 and P4 in Figure 4. Thread T1 (event generator) of process P3 is connected to thread T1 (event consumer) of process P4 via the socket descriptor *sd*.

#### 5.1.2. Event Consumers—Event-Driven Programs

**Figure 5:**
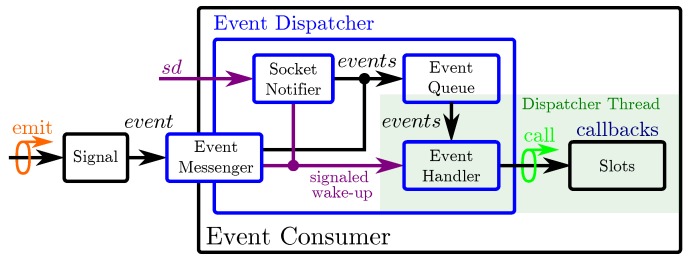
An event dispatcher combined with the signal-slot principle facilitates the connection of event generators and consumers within processes; an event handler implementing the signaled-wakeup principle dispatches events on their occurrence and drives the processing of events in the event consumer; this architecture allows multi-threaded signal-slot connections and eases the development of event generators and consumers.

Event consumers are event-driven programs that handle information on the arrival of new events. Therefore, event consumers have to wait for events and stay inactive until events arrive. During their inactivity, event consumers pause their computations and yield the timeslices of all their threads. They then resume on the arrival of events. The yielding and resuming of threads is most efficient when triggered by the signals of the operating system. Therefore, we connect event generators to these signals, see Section 5.1.1, and utilize the SWP to wait until a signal is triggered. As a result, the event consumers become only active and handle information when novel information arrives. After handling the novel information, the event consumers may, as a result, create new events that wakeup other event consumers. Thread 1 of process P3 in Figure 4 depicts such an event consumer. The realization of event consumers can be eased by utilizing event dispatchers that wait for the activity of signals and then call the appropriate callback functions (event handlers) associated to the activity of a signal (event), see Figure 5. In this setup, the handling of events takes place in callback functions. Event dispatchers can additionally implement event queues to increase the processing efficiency of agglomerated events, i.e events that occurred almost at the same time.

### 5.2. Event Decoding

Event decoders realize the bridge between the event-driven information handling system of a LASS and CDSs. At first glance decoding events is not reasonable at all. However event decoding can be seen as a compromise to utilize state-of-the-art clock-driven algorithms that have not been adapted yet for event-driven applications, see Section 3.6. Especially real-time low-level control for actuation is often provided by third parties and cannot be easily modified. In general, guaranteeing the stability of state-of-the-art controllers depends on theory that assumes that the Nyquist-Shannon sampling theorem is fulfilled at all times. This issue is addressed in event-driven control [34] that for itself is an emerging new research field. Nevertheless, up to the near future, EDSs will need to be combined with CDSs and interfaces between both systems will be required. The bridge from CDSs to EDSs are the event generators discussed in Section 3.4. The bridge from EDSs to CDSs are the event decoders that will be detailed in this section.

#### 5.2.1. Realizing Event Decoders

**Figure 6:**
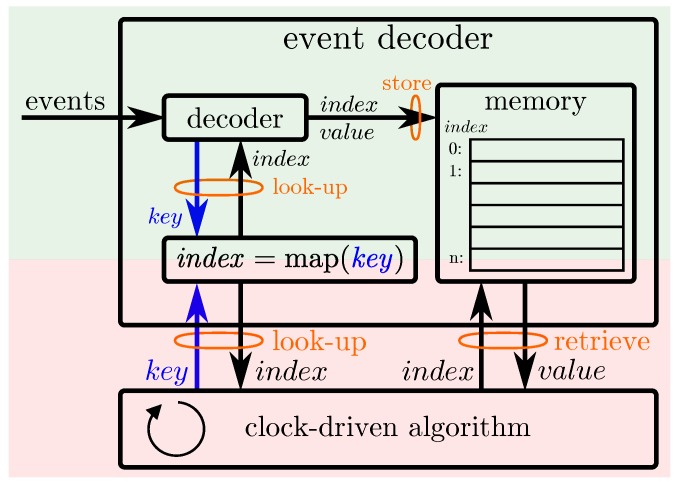
The event decoder provides an interface to transfer information from the event-driven domain (green background) to the clock-driven domain (red background).

The realization of an event decoder has to provide a synchronous interface for accessing information that is updated on the arrival of new events, see Figure 6. We propose to realize the domain crossing from event-driven to CDSs by providing two completely different interfaces to a shared memory block. The event-driven interface employs the decoder to decode events to keys and values, then utilizes a fast mapping mechanism to lookup the memory index of a key. The key, the event decoder decodes of an event, depends on its implementation and type of the event. The key could be the event ID, identifying the sensor the event originated, or the skin cell event ID, identifying the skin cell the event originated. The decoder could also combine the skin cell event ID with the sensor mask and compute the global event ID of the skin cell sensor, see Figure 3. After mapping the key to an index, the decoded value is stored in the correct memory location, see Figure 6. We detail the selection of an fast mapping mechanism for event decoders in Section 5.2.3. The clock-driven interface on the other hand provides access to the shared memory block. Since the information is stored in a contiguous memory block, clock-driven algorithms can access and re-sample information at any time and loop through all memory locations with low performance penalties. Following these examinations, we successfully realized an event decoder for our event-driven LASS. This event decoder has been extensively utilized in applications that take advantage of our event-driven e-skin and combine it with state-of-the-art control algorithms [83,86,87,88,89].

#### 5.2.2. Efficiency of Hybrid Systems

Hybrid systems containing clock-driven and event-driven components outperform pure CDSs. The overhead of the event decoder is comparable to the packet decoding in pure CDSs which also requires the mapping from skin cell IDs to indexes. Actually, on average, the event decoder outperforms the packet decoder of CDSs since its decoding and mapping resides in the event-driven domain which is less active than the clock-driven domain. Consequently, even if all event consumers are clock-driven, an event-driven e-skin system outperforms a pure CDS, as demonstrated in our evaluations in References [39,40].

#### 5.2.3. Fast Mapping Mechanisms

The fast mapping between event IDs and memory indexes can be realized with associative arrays. Associative arrays realize a fast mapping of keys (IDs) to values (indexes) and can employ different mapping techniques [90,91] such as direct addressing, self-balancing linear search trees, and hash tables, see Figure 2. These mapping techniques exhibit different advantages and disadvantages, see Table 2.

*Direct addressing* is the fastest possible mapping at the cost of high memory demands. Since the address space for event keys is huge (event keys are usually grouped in meaningful subspaces) but only sparsely occupied, direct addressing is not a suitable option for LASSs.

*Self-balancing binary search trees* (SBBST) require much less memory space than direct addressing and offer a constant lookup complexity that depends on the height of the tree, see Table 2. Consequently, SBBSTs provide fast mapping for small sets of keys. A lookup in a map with 1000 keys for example has only the complexity of 11 comparisons.

*Hash tables* provide on average a better lookup complexity than SBBST. However, finding a good hash function is essential to avoid collisions, that is, the hash function maps two or more keys to the same bucket (set of indexes). In worst case, all keys are mapped to the same bucket and the lookup complexity collapses to the performance of sequential searching, see Table 2. Thus, the definition of a good hash function depends on the distribution of keys, and if this distribution is known in advance, then the worst case can be avoided. Hash tables provide a good mapping performance for large key sets, but are less suitable for small key sets, where the overhead of the hash function and the accessing of scattered memory significantly reduces its lookup performance.

In summary, event decoders in LASSs with up to 1,000 skin cells should employ SBBSTs, since the minor lookup complexity advantage of hash tables cannot justify the disadvantage of finding a good hash function and the penalty of scattered memory. Nevertheless, event decoders in LASSs with ten-thousands of skin cells will significantly profit from hash tables.

## 6. Results

This section connects the background, theory, and realization principles towards event-driven LASSs introduced in this work.

Our first realization of an e-skin system focused on providing a scalable, flexible, and robust platform with the multi-modal sensing capabilities to enable applications requiring tactile sensation similar to the human sense of touch [29,82]. Although, the early realization follows the principles of modularity and self-organization, it reaches its limits in large-area applications. Extending these initial works with the event-driven principles for LASSs presented in this work (Section 3, Section 4 and Section 5). Hence, we succeeded in the realization of a new LASS that is low-latency while being computationally efficient [38,39,40]. Table 3 relates the challenges of LASSs with the implemented design and realization principles, and then summarizes and highlights the impacts of the principles on the implementation. For example, modularity reduces the number of connection at least by a factor of 50, or the event-driven system effectively reduces the communication traffic by around 90%.

The implementation of our event-driven LASS has been first verified on our robot platform TOMM [83,92]. TOMM has both of its arms and grippers covered with e-skin. The validation of the e-skin has been performed with one of TOMM’s UR5 arms. This experimental approach proved as good trade-off since the UR5 arm is covered with a reasonable amount of skin cells (253 skin cells, 2024 sensors) while a CDS is at the same time still capable to handle the intermediate amount of tactile information. The ability to perform experiments in clock-driven and event-driven mode with identical systems enables fair and comprehensive evaluations and comparisons of EDSs with their clock-driven counterparts [39]. Actually, the design of a fully clock-driven control system operating with clock-driven tactile information of 253 skin cells is still feasible. This allows us to fully assess the performance of a complete EDS with sophisticated event consumers and controllers in meaningful applications [93].

The effectiveness of the EDS in comparison to its clock-driven counterpart in various experimental evaluations and applications [38,39,40,83,87,88,89,93], including control [39,87,88,89,93], can be assessed by analyzing the indicators: 1) the *network traffic* between the deployed skin system and the information handling system; and 2) the *CPU load* of the perception module in the information handling system. While deeper analysis and evaluations with many additional indicators (e.g., performance of control) have been performed previously, this work focuses solely on the indicators network traffic and CPU load to provide a comprehensive overview of all results in the context of validating the presented design and realization principles.

**Figure 7:**
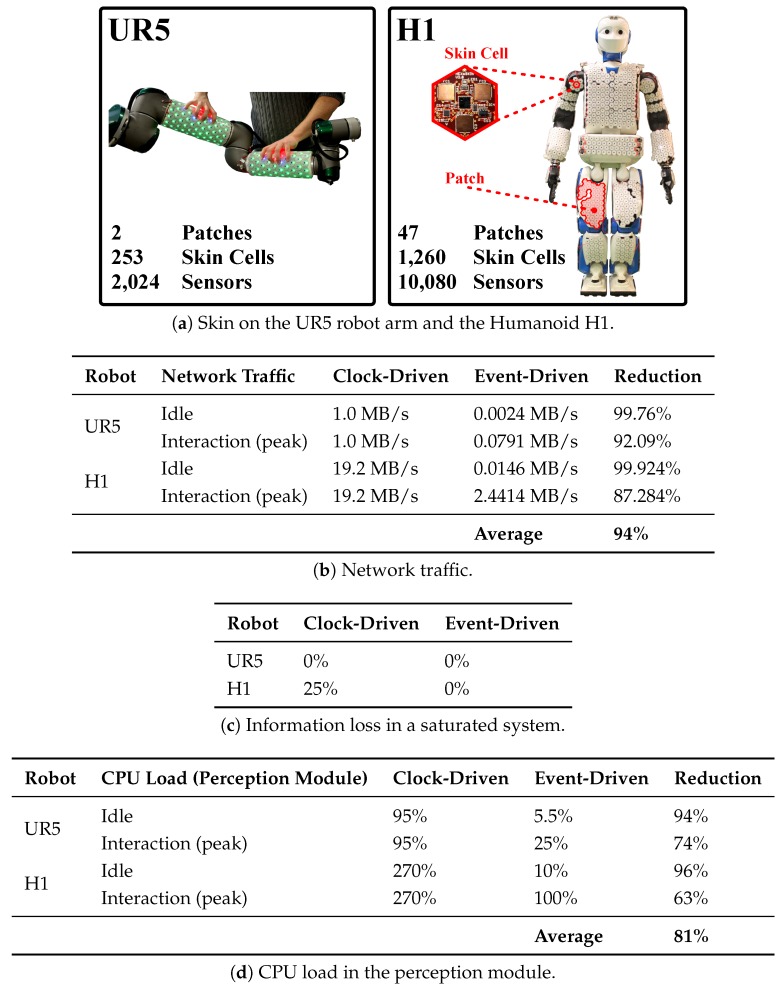
Network traffic and CPU load of the perception module of our e-skin system on one UR5 arm with 253 deployed skin cells (125 Hz sampling clock) [93] and on H1 with 1260 deployed skin cells (250 Hz sampling clock) [40]; the idle e-skin system does not register any tactile interactions; any interaction with the e-skin generates information rate peaks that mirror to event rate peaks and thus to peaks with the network traffic or the CPU load; tactile interactions vary during our experimental setups; they range from stroking, hammering, reactive control (contact avoidance) [39,93] to covering the whole humanoid with a cloth [40], touching the robot through the cloth, and shaking the cloth; here, we ensure the comparability in the overview of our evaluations by focusing on the measured peaks and treating them as worst case measurements; the evaluation presented in Reference [40] is more detailed and additionally provides stochastic evidence; the perception module causes CPU load for providing the information of the network traffic to the higher-level information handlers; 100% CPU load equals the saturation of one CPU core in simultaneous threading mode, the CPU loads measured in the clock-driven setup of H1 are lower than expected since the information handling system is totally saturated and can not assign more CPU time to the perceptive information handling; the CDS of H1 effectively handles 25% less information than required since 25% of the information is lost.

The evaluation results of the e-skin system on the UR5 arm are presented in Figure 7. Both indicators show a substantial reduction for the event-driven approach in comparison to the clock-driven reference. While both indicators are constant in CDSs, even when the system is idle and the information rate is zero, in EDSs, both indicators depend on the information rate or respectively on the event rate.

Figure 7 additionally summarizes our evaluation results for the scaled-up event-driven e-skin system we later deployed on our humanoid robot H1 [40,83]. This large-area e-skin system incorporates 1260 skin cells and 10,080 sensors [83] on 0.87 m${}^{2}$, fitting well into the field of large-scale tactile sensing where other works [28,94] at most deploy 2,208 sensors of one modality on an area of around 0.07 m${}^{2}$. The evaluation of the LASS on H1 demonstrates that the superior efficiency of the event-driven is now required to avoid the loss of information. In clock-driven mode, the LASS on H1 overloads the information handling system and around one quarter of the tactile information is lost. The clock-driven LASS on H1 already fails in perceptive information handling and a further behavioral information handling to realize applications is definitely not feasible. Thus, effective LASSs are only feasible in event-driven setups.

## 7. Conclusions

This work presented the foundations for realizing, designing, and understanding large-area event-driven e-skin systems for effective applications. Homogenizing the perspectives on event-driven systems of the different research fields and consolidating the challenges of large-area skin systems provided the basis for assessing existing event-driven approaches. This assessment identified the send-on-delta principle (SoDP) as the most applicable method for large-area skin systems (LASSs). The send-on-delta principle offers a high system flexibility combining well with the measures for improving the deployability of large-area skin systems. The subsequent presentation of designs, supported with the previously consolidated theory, include a novel set of guidelines for tuning the novelty-threshold of event generators, modular event generators, event decoders, and a novel systematic design approach towards realizing event-driven information handling systems on standard computing systems. The presented design principles have been validated by outlining their impacts on our large-area skin implementations and by consolidating their experimental results. The experimental evaluations compared the event-driven large-area skin system with the networking and computational performance of its clock-driven counterpart. The event-driven large-area skin system outperforms the clock-driven one on average by a reduced network load of 94% and a reduced CPU load of 81%. In its large-area setup with 10,080 sensors, the clock-driven large-area skin system computationally saturated the computer system and could not operate without information loss (25% of all information was lost). Whereas the same system driven by events did not saturate and experienced very little losses, even under the same experimental condition with major tactile stimulation, that is, covering a humanoid robot with a cloth and moving and pressing the cloth, in total only 80 events (⋘0.1% information loss). Although, both systems observed information losses, these losses are largely originating from overflowing queues. Computer systems store arriving information in queues until the operating system schedules a thread to retrieve the information. Thus, when a computer system saturates, that is, more information arrives than can be processed per time instance, then these queues overflow and information is lost. Consequently, the continuous saturation of a clock-driven system causes continuous information loss. Rather than saturation, event rate peaks can cause sporadic information losses in event-driven systems. Event-driven computer systems lose information when more events arrive at the same time than events are fitting into the queues. The information loss in a saturated clock-driven computer system cannot be mitigated, but the information loss in event-driven computer systems can be reduced by increasing the queue sizes at the cost of an increased latency at event rate peaks. Overall, the presented foundations lead to scalable, efficient, and flexible e-skin systems, capable of handling large amounts of information, and improving the feasibility of complex large-area tactile applications, for instance in robotics.

## Figures and Tables

**Table 1:** Comparison of different information encoding, representation, and transmission systems.

System	Connections	Bandwidth	Arbitration	Representation	Encoding	Decoding	Bit Rate	Latency	Temporal Precision
Nerve Bundle	thousands of nerve fibers	low, ≤1 kEvents/s per fiber	none	binary action potential (1 bit) asynchronous events	neural codes	complex	very low	medium	extremely high
Clock-Driven	few, serial bus	very high, >10 MSamples/s	standard network routing, time-multiplexed, flexible	samples/packets of samples (many bits) synchronous stream	absolute values	none	constantly very high	medium/high	low
AER	many, parallel bus	high, ∼100 MEvents/s	complex handshaking, time-multiplexed, inflexible	address (address bits) asynchronous events	neural codes	complex	low	low	high
Serial AER	few, serial bus	high, ∼5 MEvents/s	complex handshaking, time-multiplexed, inflexible	serialized address asynchronous events	neural codes	complex	low	higher than AER	lower than AER
ACES	1 wire, upto 138 k sources	low, ∼ 1 kEvents/s	none	pulse signature asynchronous events	neural codes	very complex, not event-driven	low	medium	very high
**SoDP**	**few**, serial bus	medium, >1.5 MEvents/s	standard network routing, time-multiplexed, **flexible**	event packet (sample bits) asynchronous events	absolute values	**simple**	medium	**low**	medium

**Table 2:** Average and Worst Case search complexity, and memory requirements for *n* elements with *m* bit keys.

Data Structure	Avg. Search	WC. Search	Space
direct addressing	O(1)	O(1)	$2^{m}$
self-balancing binary search tree	O(logn)	O(logn)	*n*
hash table	O(1)	O(n)	*n*

**Table 3:** Challenges, designs, and their impacts on scalable large-area skin systems (LASSs).

Challenges	Designs/Realizations	Impacts/Results (UR5 + H1)
Reliability/Robustness (C-1)	modules: hexagonally shaped skin cells local processing capabilities at skin cells redundant meshed network of skin cells dynamic routing	upto N + 3 redundancy in skin patches automatic online failure recovery within less than 50 ms
Deployability (C-2)	hierarchical modular structure (cells, patches, segments) self-organizing network	no manual construction of communication trees flexible addition/removal of modules (cells, patches, segments) eased deployment of: – UR5: 2 patches instead of 253 cells (2024 sensors) – H1: 47 patches in 12 segments instead of 1260 cells (10,080 sensors)
Wiring (C-3)	hierarchical modular structure modular interfaces	huge reduction of wire count by a factor >50 reduction of connections from: – UR5: 253 (2024) point-to-point to 4 patch connections – H1: 1260 (10,080) point-to-point to 12 interface connections
Sensor Localization (C-4)	2D information of self-organized modular structure rotation measurement between cells automatic 3D surface reconstruction of patches	automatic self-calibration of relative sensor locations huge reduction of manual localization tasks by a factor >80 manual/semi-automatic localization of: – UR5: 2 patches instead of 253 cells (2024 sensors) – H1: 47 patches instead of 1260 cells (10,080 sensors)
Efficient Information Handling (C-5, C-6)	modular SoDP with event generators in skin cells efficient event decoders to bridge to clock-driven algorithms event-driven information handling framework exploiting the asynchronous scheduling capabilities of standard operating systems	the system scales well from 253 to 1260 cells EDS is the key for information handling in LSSSs: – loss-less information handling (H1: 25% loss when clock-driven) – effective reduction of the data rate by around 90% – efficient information handing: computational load reduced by around 60%

## Abbreviations

The following abbreviations are used in this manuscript:

e-skin	electronic skin
LASS	large-area skin system
EDS	event-driven system
CDS	clock-driven system
NDS	novelty-driven system
AER	address-event representation
SoDP	send-on-delta principle
ACES	asynchronous encoded skin
EP	endpoint
SWP	signaled wakeup principle
SBBST	self-balancing binary search trees
PC	personal computer
IC	integrated circuit
VLSI	very large scale integration

## Appendix A. Requesting and Yielding Computation Time

Programs, whether they are clock- or event-driven, often run in conditions where they have to wait. They need to wait until information is available or can be forwarded to the next stage. Clock-driven programs may need to wait to synchronize to a desired sampling rate, while event-driven programs may need to sleep until novel information arrives. Ideally, all programs should wait in such a way that the operating system can switch context, that is, pause the current task and schedule another process/thread as soon as a program enters a waiting condition. In this way no computation time is wasted while waiting. Programs can usually wait in three different ways, by

1. Busy-Waiting,
2. Timed-Waiting, and
3. Signaled-Wakeup.

*Busy-Waiting*, often also called active waiting or polling, describes a program that waits for a condition/flag, by repeatedly reading it until it is true, see Algorithm A1.

**Algorithm A1** Busy-Waiting
**while***flag* == false **do** # do nothing, waste time **end while**

The thread executing this program never notifies the operating system that it is waiting and that it could yield its allotted timeslice for other, more important tasks. Actually, a thread that never yields is considered as *greedy*, since the operating system assigns this thread as much computation time as possible and the thread consumes all of it, if needed or not. A greedy thread never stops before it consumed its timeslice. Busy-Waiting is wasting the limited resource computation time, is thus extremely inefficient, and should be avoided.

*Timed-Waiting* is more efficient, since the thread notifies the operating system how long it can manage without computation time. In contrast to Busy-Waiting, Timed-Waiting checks the flag/condition with a rate defined by the sleep time $T_{s}$, rather than checking it as fast as possible, see Algorithm A2.

**Algorithm A2** Timed-Waiting
**while***flag* == false **do** $\mathrm{sleep}(T_{s})$ # yield and reschedule after $T_{s}$ **end while**

The sleep function yields the timeslice of the thread and defines the time when it is scheduled next. Timed-Waiting is not wasting computation time and is an efficient waiting principle for clock-driven programs with a defined sampling frequency.

*Signaled-Wakeup* is realized in programs that wait for signals of the operating system rather than for flags. These signals can be defined and triggered by other processes/threads, or are provided by the operating system. The operating system can provide signals that are triggered by hardware interrupts, for example, the arrival of network packets, and so forth. Exploiting the Signaled-Wakeup principle in programs is even more efficient than Timed-Waiting. Employing Signaled-Wakeup, a thread yields and notifies the operating system that it is waiting for a signal, see Algorithm A3.

**Algorithm A3** Signaled-Wakeup
# do something select(signal) # yield and reschedule on signal # continue

Rather than repeatedly reassigning timeslices for the program to read a flag, or check a condition, as in Timed-Waiting, the operating system only returns to the thread on the occurrence of the signal. Obviously, the proper exploitation of the Signaled-Wakeup principle enables us to realize fast event-driven information handling in standard computing systems with low waiting overhead.
